# Hematological Findings and Clinical Severity in Pediatric Patients with COVID-19

**DOI:** 10.4274/tjh.galenos.2021.2021.0488

**Published:** 2021-12-07

**Authors:** Pathum Sookaromdee, Viroj Wiwanitkit

**Affiliations:** 1 Private Academic Consultant, Bangkok, Thailand; 2 Patil University, Pune, India

**Keywords:** Hematology, Severity, COVID-19, Pediatric

## To the Editor,

We would like to share some ideas on “Can Hematological Findings of COVID-19 in Pediatric Patients Guide Physicians Regarding Clinical Severity?” [1]. Arıkan et al. [1] concluded that the “neutrophil-to-lymphocyte ratio (NLR) was higher in severe/critical cases… Red cell distribution width (RDW) statistically significantly increased in severe cases”. We agree that basic hematological parameters might be good indicators for monitoring the severity of many medical problems. Regarding coronavirus disease-19 (COVID-19), NLR and RDW might or might not be useful as predictive parameters for severity in children with COVID-19. RDW might not be useful in many settings. In our setting in Southeast Asia, there is a very high incidence of thalassemia. There are also many pediatric cases of COVID-19. Since these children usually have high RDW as a background hematological finding [2], RDW is not useful for the prediction of COVID-19 severity in these cases. Therefore, RDW is not a useful predictive parameter in any setting with a high incidence of thalassemia. The NLR ratio might be a better predictive parameter. However, it is necessary to set a population-specific reference value for clinical application [3].
