# Supplementary material for: Hematological Findings and Clinical Severity in Pediatric Patients with COVID-19
Source: Turk J Haematol. 2021 Dec 7;38(4):329–30. doi: 10.4274/tjh.galenos.2021.2021.0488 (PMC8656126; doi:10.4274/tjh.galenos.2021.2021.0488)
Supplement: Related texts

# REPLY FROM THE AUTHORS

We thank the author for their interest in our manuscript, and we acknowledge their concerns regarding  use of red cell distribution width (RDW)  in clinical diagnosis of  severity  of COVID-19 infected children in places with very high incidence of thalassemia like  Indochina. While we agree that pediatric children usually have a high RDW as a background hematological disease, in our study none of the patients had thalassemia as underlying disease. And published studies support usage  hematological  parameters including RDW in COVID-19 infected cases.

In a published study, a progressive increase of RDW was observed with advancing COVID-19 severity. Also in multivariate analysis, elevated RDW was associated with 9-fold increased odds of severe COVID-19 [1].

In an other published study, RDW was found to be a prognostic predictor for patients with severe COVID-19, and  authors concluded that the increase in reticulocyte  may contribute to elevated RDW [2].In a study conducted in 1641 patients, authors concluded that elevated RDW at the time of hospital admission and an increase in RDW during hospitalization were associated with increased mortality risk for patients with COVID-19 [3].

In our findings, the NLR ratio was higher in severe/critical cases compared to cases of asymptomatic, mild, and moderate severity. In a study  conducted in 245 COVID-19 patients multivariate analysis demonstrated that there was 8% higher risk of in-hospital mortality for each unit increase in NLR (odds ratio [OR] =1.08; 95% confidence interval [95% CI], 1.01 to 1.14; P=0.0147) [4].

Finally  we conclude  that hematological parameters of COVID-19 infected pediatric cases may alert physicians about clinical severity. But hematological markers especially RDW should be used correctly as patient-based approach in COVID-19 infected pediatric patients.

**Kamile Ötiken Arıkan, Şahika Şahinkaya, Elif Böncüoğlu, Elif Kıymet, Ela Cem, Aybüke Akaslan Kara, Nuri Bayram, İlker Devrim**

## References

[1] Henry BM, Benoit JL, Benoit S, Pulvino C, Berger BA, Olivera MHS, Crutchfield CA, Lippi G (2020). Red Blood Cell Distribution Width (RDW) Predicts COVID-19 Severity: A Prospective, Observational Study from the Cincinnati SARS-CoV-2 Emergency Department Cohort. Diagnostics (Basel).

[2] Wang C, Zhang H, Cao X, Deng R, Ye Y, Fu Z, Gou L, Shao F, Li J, Fu W, Zhang X, Ding X, Xiao J, Wu C, Li T, Qi H, Li C, Lu Z (2020). Red cell distribution width (RDW): a prognostic indicator of severe COVID-19. Ann Transl Med.

[3] Foy BH, Carlson JCT, Reinertsen E, Padros I Valls R, Pallares Lopez R, Palanques-Tost E, Mow C, Westover MB, Aguirre AD, Higgins JM (2020). Association of Red Blood Cell Distribution Width With Mortality Risk in Hospitalized Adults With SARS-CoV-2 Infection. JAMA Netw Open.

[4] Liu Y, Du X, Chen J, Jin Y, Peng L, Wang HHX, Luo M, Chen L, Zhao Y (2020). Neutrophil-to-lymphocyte ratio as an independent risk factor for mortality in hospitalized patients with COVID-19. J Infect.
